# Microbial Ecology and Evolution Are Essential for Understanding Pandemics

**DOI:** 10.1128/mBio.02144-21

**Published:** 2021-09-28

**Authors:** Megan E. Frederickson, Aspen T. Reese

**Affiliations:** a Department of Ecology & Evolutionary Biology, University of Toronto, Toronto, Canada; b Department of Biological Sciences, University of California, San Diego, San Diego, California, USA

**Keywords:** COVID-19, microbial ecology, microbial evolution, microbiome

## Abstract

Ecology and evolution, especially of microbes, have never been more relevant than in our global fight against SARS-CoV-2, the virus that causes COVID-19. Understanding how populations of SARS-CoV-2 grow, disperse, and evolve is of critical importance to managing the COVID-19 pandemic, and these questions are fundamentally ecological and evolutionary in nature. We compiled data from *bioRxiv* and *medRxiv* preprint abstracts and US National Institutes of Health Research Project grant abstracts to visualize the impact that the pivot to COVID-19 research has had on the study of microbes across biological disciplines. Finding that the pivot appears weaker in ecology and evolutionary biology than in other areas of biology, we discuss why the ecology and evolution of microbes, both pathogenic and otherwise, need renewed attention and investment going forward.

## COMMENTARY

An adage says there is nothing certain in life except death and taxes, but we disagree. If the COVID-19 pandemic has taught us anything, it is that there is nothing certain in life except exponential growth and adaptation.

Populations grow exponentially unless something keeps them in check—usually, they eventually run out of resources. For SARS-CoV-2, this would mean having no more susceptible hosts. But even as resources grow scarce, populations get better at exploiting them. New genetic variants arise that leave more descendants, and those variants spread within populations and to new regions, just as SARS-CoV-2 variants have done (1).

How populations grow, spread geographically, and adapt are fundamentally ecological and evolutionary questions. But although tracking SARS-CoV-2 population growth, geographic spread, and variants has been a major focus of scientific research, government funding, and media coverage during the COVID-19 pandemic (2, 3), this tracking is often presented absent a broader understanding of ecology or evolutionary biology. Here, we argue that to better understand and predict these and related processes requires sustained basic research into the ecological and evolutionary dynamics of microbes generally, be they pathogens or not.

It was not that long ago that beneficial microbes, not pathogens, were in the limelight, and the microbiome was one of the hottest topics in biology. In the years leading up to the COVID-19 pandemic, biologists had gradually moved away from thinking of microbes primarily as disease-causing “germs” and began to appreciate the diverse communities of nonpathogenic microbes that often colonize hosts—i.e., the host-associated microbiome (4, 5). In microbiome science, as in much work on SARS-CoV-2, the fundamental questions are also often inherently ecological or evolutionary in nature, as we highlight in Table 1 (see also references 6–8). Answering these questions is necessary and urgent, both for controlling the spread of SARS-CoV-2 and other novel pathogens and for developing new microbial therapies and applications that leverage the potential of beneficial microbiota (9).

**TABLE 1:** Similar ecological and evolutionary questions pertain to the study of SARS-CoV-2 and to host-associated microbiomes^*a*^

Fundamental ecological or evolutionary question	SARS-CoV-2	Microbiomes
How do microbes move into new host species?	To help identify the source of SARS-CoV-2, Zhou et al. (17) sequenced viruses from 411 bat samples collected in Yunnan, China, between May 2019 and November 2020 and found four new coronaviruses related to SARS-CoV-2.	Free-living microbes can, and often do, evolve to be host-associated (32), and microbes vary substantially in how often they shift into new host species. For example, fewer than 20% of microbial species found in nonhuman primates also occur in humans (33), but some microbial species, such as Lactobacillus reuteri, are found in the microbiomes of many vertebrate hosts (34).
How do individual hosts acquire microbes?	Kutter et al. (35) showed that SARS-CoV-2 can be transmitted between ferrets in cages connected only by an airway duct. Genomic and epidemiological sleuthing by Eichler et al. (27) documented a probable case of aerosol transmission of SARS-CoV-2 without direct person-to-person contact in a quarantine hotel.	Maternal transmission plays a large role in seeding the human infant gut microbiota, but tracking rare single nucleotide variants in metagenomes showed that environmental acquisition also occurs early in life (36).
What limits a microbe’s spread: abiotic factors such as climate, or biotic factors such as host density, behavior, or traits?	Early in the pandemic, low caseloads in the Southern Hemisphere and tropical regions led some to speculate the virus was constrained by climate. Baker et al. (37) used epidemiological models to show that while climate may be limiting to endemic infections, it plays little role in shaping dynamics during the pandemic stage. Rader et al. (38) found that climate was associated with the peakedness of epidemic cases but that crowding explained more about the spread and total number of cases in cities.	A meta-analysis of over 15,000 samples representing 654 host species showed strong climatic signals in external microbiomes while host physiology and behavior (e.g., diet) were more important for structuring internal microbiomes (39).
How often do new variants or strains arise? How, and how fast, do microbes adapt to hosts?	Most infections do not present sufficient opportunity or selection for new variants to arise, but multiple variants have arisen with mutations in the spike protein, some with identical mutations suggesting that selection is occurring for that mutation (1, 23, and references therein).	Garud et al. (40) demonstrated evidence of human gut microbial local adaptation driven by nucleotide variants, gene gains or losses, and recombination over short time scales but suggested that replacement by novel strains from transmission matters more over longer time scales.
Do microbes evolve to be more beneficial or more virulent?	Yurkovetskiy et al. (41) documented an early SARS-CoV-2 spike protein variant which was more infective of human cells and quickly swept the circulating viral population.	Either increased host benefits or increased pathogenicity can evolve in host-associated microbes (42–44). Using experimental evolution, Batstone et al. (43) found that beneficial bacteria evolve to provide more benefits to their local hosts. In contrast, experimental evolution of a close relative of Mycobacterium tuberculosis identified mutations that enhance persistence and immune resistance in hosts, setting the stage for the emergence of pathogenicity (44).

a We highlight recent research on five of the most pressing questions regarding SARS-CoV-2 and microbiomes. Note that the authors of these studies did not always take an explicitly ecological or evolutionary perspective.

**FIG 1:**
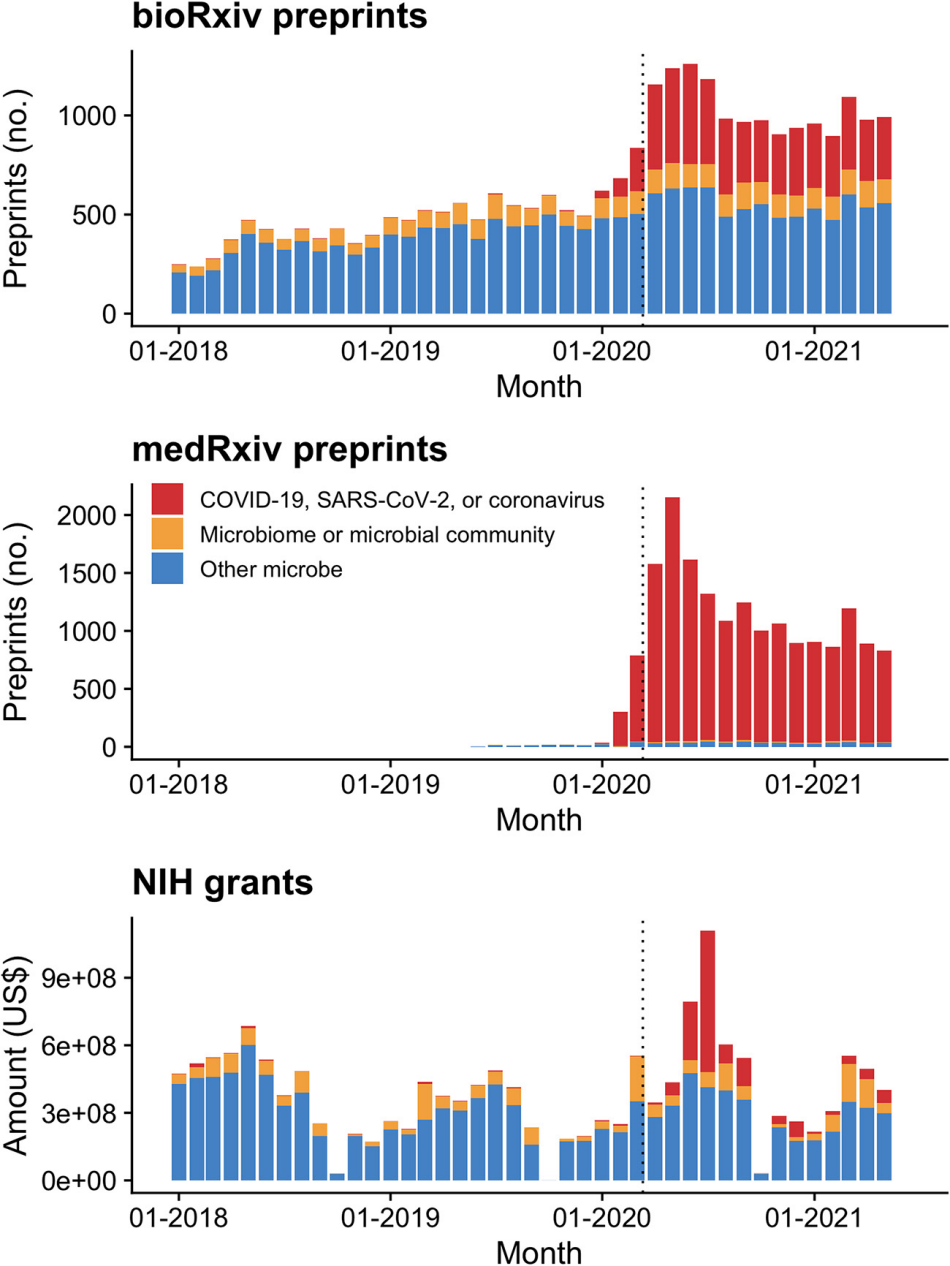
Number of *bioRxiv* and *medRxiv* preprints and total amounts of US National Institutes of Health Research Project Grants per month with microbe-related terms in their abstracts, January 2018 to May 2021. To visualize the scale of the scientific community’s mobilization to understand SARS-CoV-2 and COVID-19, we analyzed *bioRxiv* and *medRxiv* preprint data and US National Institutes of Health grant data from before and during the COVID-19 pandemic. We performed case-insensitive searches for “COVID,” “SARS-CoV-2,” “2019-nCoV,” or “coronavirus” (red) in preprint and grant abstracts and compared the results to search terms related to microbiome science (“microbiome” or “microbial community,” orange) or other microbial search terms (singular and plural forms of “microbe,” “bacteria,” “fungi,” “virus,” or “archaea,” labeled “other microbe,” blue). Dotted lines are 11 March 2020, the date when the World Health Organization declared COVID-19 a pandemic.

Despite surging interest in microbiome science over the last few years, starting in March 2020, preprints and grants on COVID-19 eclipsed much other microbial research (Fig. 1). It seems likely that SARS-CoV-2 is now the most studied microbe on Earth (Fig. 1; see also Fig. S1 in the supplemental material and reference 3). It is equally clear that coronaviruses had been neglected before early 2020, despite the warnings we had with the SARS and MERS epidemics in 2003 and 2012, respectively (10, 11); prepandemic studies of coronaviruses appear as the miniscule red bars before 2020 in Fig. 1.

10.1128/mBio.02144-21.1 FIG S1 Number of *bioRxiv* preprints per month with microbe-related terms in their abstracts, by author-tagged disciplinary categories, January 2018 to May 2021. We performed case-insensitive searches for “COVID,” “SARS-CoV-2,” “2019-nCoV,” or “coronavirus” (red) in *bioRxiv* preprint abstracts and compared the results to search terms related to microbiome science (“microbiome” or “microbial community,” orange) or other microbial search terms (singular and plural forms of “microbe,” “bacteria,” “fungi,” “virus,” or “archaea,” labeled “other microbe,” blue). Shown are all disciplinary categories in the *bioRxiv* database with at least 25 preprints on COVID-19, SARS-CoV-2, or coronaviruses; excluded categories (i.e., categories with fewer than 25 preprints on COVID-19, SARS-CoV-2, or coronaviruses) are animal behavior and cognition, cancer biology, clinical trials, developmental biology, epidemiology, paleontology, physiology, plant biology, synthetic biology, and zoology. Clinical trials and epidemiology have very few preprints submitted to *bioRxiv* since June 2019, when *medRxiv* was founded; preprints in these disciplines have been overwhelmingly submitted to *medRxiv* during the COVID-19 pandemic (Fig. 1). Dotted lines are 11 March 2020, the date when the World Health Organization declared COVID-19 a pandemic. Download FIG S1, PDF file, 0.02 MB. Copyright © 2021 Frederickson and Reese. 2021 Frederickson and Reese. https://creativecommons.org/licenses/by/4.0/ This content is distributed under the terms of the Creative Commons Attribution 4.0 International license.

**FIG 2:**
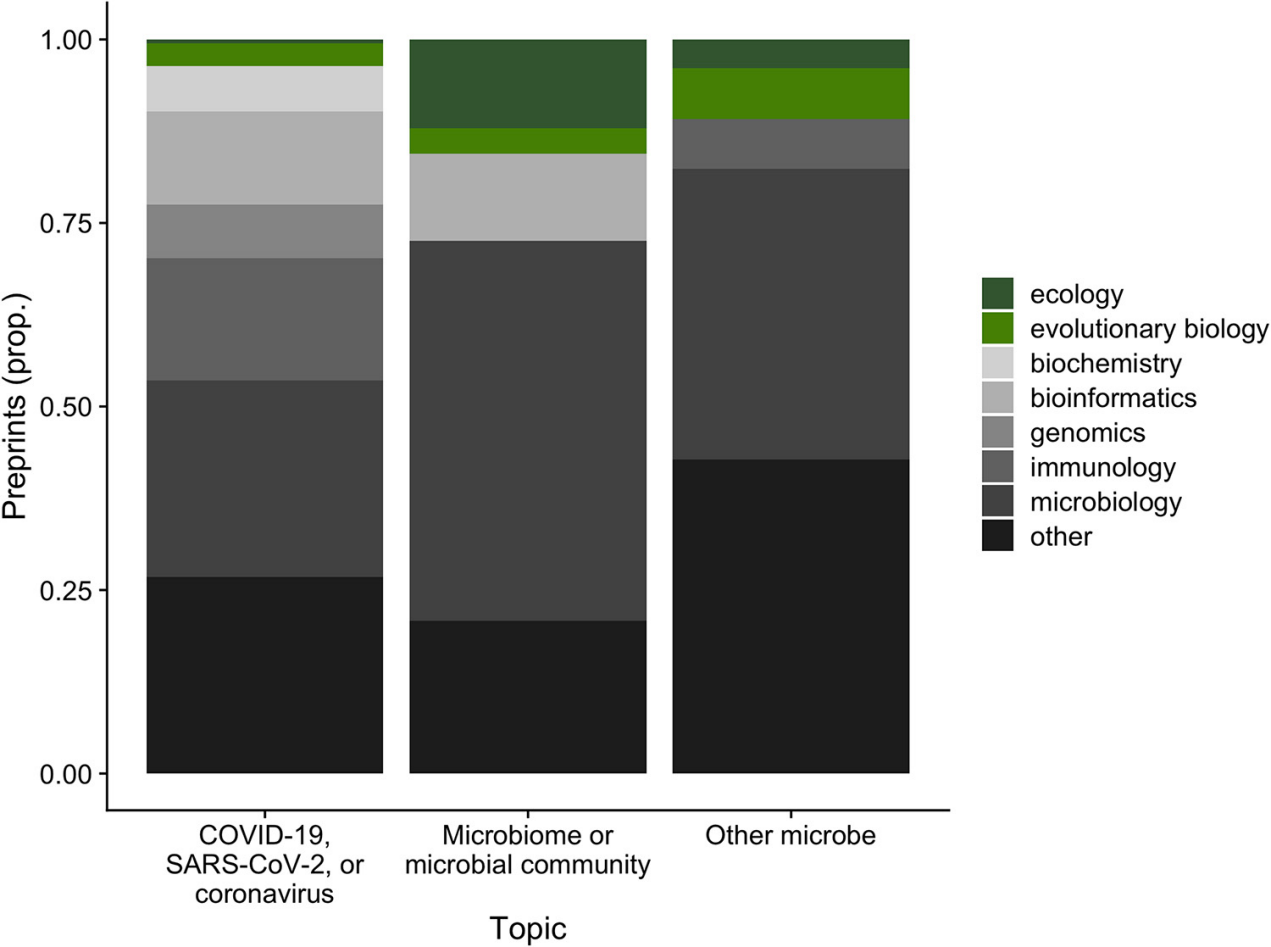
Proportion (prop.) of *bioRxiv* preprints with microbe-related terms in their abstracts submitted between January 2018 and May 2021, by author-tagged disciplinary categories. We performed case-insensitive searches for “COVID,” “SARS-CoV-2,” “2019-nCoV,” or “coronavirus” (left bar) in *bioRxiv* preprint abstracts and compared the results to search terms related to microbiome science (“microbiome” or “microbial community,” middle bar) or other microbial search terms (singular and plural forms of “microbe,” “bacteria,” “fungi,” “virus,” or “archaea,” labeled “other microbe,” right bar). Shown are the proportions of preprints in ecology (dark green) and evolutionary biology (light green), and in disciplines with at least 6% of submitted preprints for that topic (gray scale); for each topic, except for ecology and evolutionary biology, disciplines accounting for less than 6% of submitted preprints are pooled and labeled “other.”

This pivot to COVID-19 research occurred not only in fields one might expect, such as immunology and microbiology, but also in biochemistry, bioinformatics, genomics, and most other disciplinary categories in the *bioRxiv* preprint database (Fig. 2; Fig. S1). Nonetheless, the pivot to COVID-19 research appears notably weaker in ecology and evolutionary biology (Fig. 2; Fig. S1). Together, ecology and evolutionary biology account for just 3.6% of *bioRxiv* preprints on COVID-19, SARS-CoV-2, or coronaviruses, compared to 15.5% of *bioRxiv* preprints on microbiomes and microbial communities, and 10.8% of *bioRxiv* preprints on other microbes (Fig. 2). We think this represents a missed opportunity.

COVID-19 research did not emerge in a vacuum but has been profoundly shaped by previous basic, curiosity-driven research in all these fields. An example that has already gotten a lot of attention is the development of mRNA vaccines, rightly celebrated as the culmination of decades of fundamental research in molecular biology and nanotechnology (12, 13). In contrast, the influence of ecology and evolutionary biology is sometimes overlooked. The proximate source of the models widely used to predict COVID-19 case counts is the discipline of epidemiology, but epidemiological models are a special case of more general population ecological models, and it was ecologists who wrote the foundational text on modern infectious disease dynamics (14). By studying biological invasions, ecologists have developed a framework for understanding how organisms move around the world via human transportation networks, and how they establish, spread, and adapt rapidly in new environments, just as SARS-CoV-2 is doing (15). The phylogenetic tools used to study how the SARS-CoV-2 lineage “spilled over” into humans (16, 17) and to trace its spread around the globe (1, 18–21) were developed by evolutionary biologists to reconstruct the evolutionary histories of diverse plant, animal, and microbial lineages across the tree of life. The formal theoretical basis for understanding whether new variants, like Delta, are spreading because of adaptation or demographic processes (e.g., drift) also comes from evolutionary biology (22, 23), as do methods for determining the extent of purifying versus positive selection acting on the SARS-CoV-2 genome (1). And so on.

Despite intensive research into SARS-CoV-2 during the COVID-19 pandemic (Fig. 1), we still have much to learn about this virus and its interactions with both human and nonhuman hosts. Better situating SARS-CoV-2 research within ecology and evolutionary biology has the potential to offer many insights (15, 22, 23). Furthermore, the next pandemic might not be a coronavirus, so it behooves us to ramp up efforts to study diverse host-microbe interactions now (24). Researchers have begun to address the fundamental questions about SARS-CoV-2 listed in Table 1, but there are also many other open questions that would benefit from the expertise of ecologists and evolutionary biologists. For example, what limits the distribution and abundance of microbes in wild animals? For almost all except the best-studied microbes, we have no idea, but contemporary ecology offers a robust toolkit we can use to find out, and the answer is critical to understanding the risks they pose (or benefits they may offer) to humans. What might we learn from studying host behaviors that impact microbial transmission, including social distancing, in other animals (25)? Animals from primates to ants modify their social networks in ways that reduce pathogen transmission (26, 45). How do social distancing or other host behaviors change the course of pathogen versus mutualist evolution (22, 28)? How do pathogens interact with commensal or beneficial microbes in the host microbiome, and can the “right” microbiome be protective (28, 29)? How do microbes evolve as they disperse from one host species to another (and sometimes back again [23])? Should we expect SARS-CoV-2 to evolve to escape vaccines, even though microbes do not evolve vaccine resistance as readily as drug resistance (23, 30)? How do ecological and evolutionary dynamics within individual hosts affect spread among hosts, and what do within-host dynamics mean for disease progression and treatment of infected individuals (31)? We need to draw on the foundation laid by the last century of basic research in ecology and evolutionary biology, and especially on recent advances in microbial and disease ecology, and evolutionary medicine, to answer these and related questions.

As important and timely as the massive research enterprise surrounding COVID-19 is, it is also worth considering what research is currently being neglected or abandoned. We wonder, for example, whether the study of beneficial microbes and host-associated microbiomes will once again be overshadowed by research on “germs,” even though beneficial microbes are an important counterfactual to pathogens, often protect hosts against disease, and are a promising potential source of new therapies and applications (9, 28). The preprint and grant data set we assembled suggests that, to date, research and funding focused on SARS-CoV-2 have mainly been in addition to, rather than instead of, research on the microbiome or other microbial science (Fig. 1; Fig. S1). Still, as ecologists and evolutionary biologists, we know that time and resources are never infinite, and limited resources give rise to trade-offs, so this pattern will probably not continue indefinitely.

In sum, our understanding of SARS-CoV-2 population growth, geographic spread, and evolution, and thus our ability to predict and manage the COVID-19 pandemic, would benefit from greater integration among the fields of epidemiology, public health, microbiology, ecology, and evolutionary biology. Furthermore, although it may be tempting to focus exclusively on studying SARS-CoV-2 because of the urgency and scale of the COVID-19 pandemic, we should not neglect fundamental research into the ecological and evolutionary dynamics of a diversity of microbes from across the mutualism-to-parasitism continuum. These efforts may accelerate solutions to the challenges presented by SARS-CoV-2 and help prepare us for future pandemics, as we find better ways to understand, manage, and live with the certainty of exponential growth and adaptation.

### Data availability.

All analyses use publicly available preprint and grant data, and a full description of data sources and code is available at https://github.com/drfreder/pandemic-microbes.
