# Supplementary figures and images for: Microbial Ecology and Evolution Are Essential for Understanding Pandemics
Source: mBio. 2021 Sep 28;12(5):e02144-21. doi: 10.1128/mBio.02144-21 (PMC8546628; doi:10.1128/mBio.02144-21)

# bioRxiv preprints

Preprints (no.)

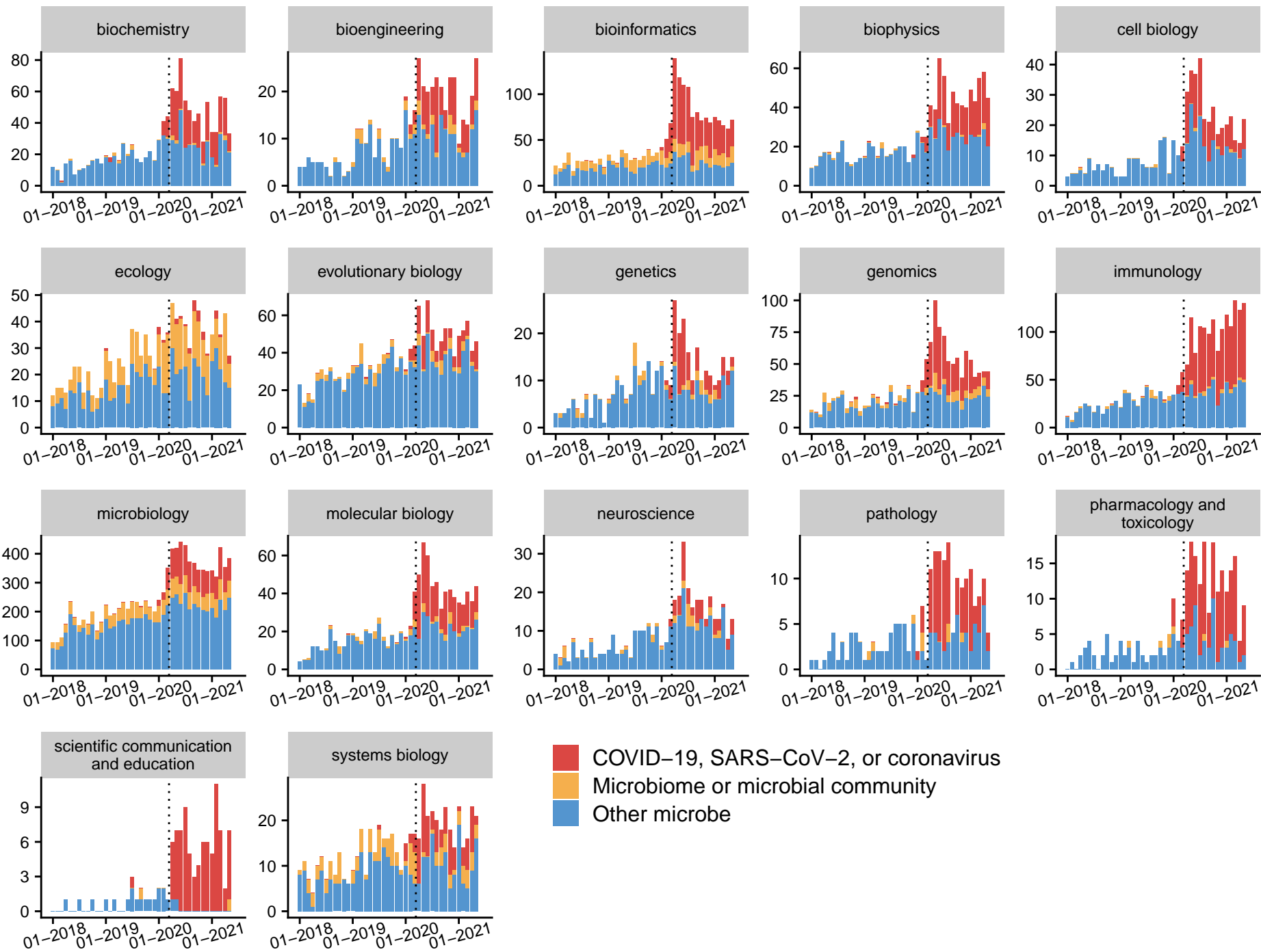

Month

Supplement: FIG S1 [file mbio.02144-21-sf001.pdf]
